# Prospective associations between adolescent mental health problems and positive mental wellbeing in early old age

**DOI:** 10.1186/s13034-016-0099-2

**Published:** 2016-06-08

**Authors:** Atsushi Nishida, Marcus Richards, Mai Stafford

**Affiliations:** Department of Psychiatry and Behavioural Science, Tokyo Metropolitan Institute of Medical Science, Kamikitazawa 2-1-6, Setagaya, Tokyo, 156-8506 Japan; MRC Unit for Lifelong Health and Ageing at UCL, 33 Bedford Place, London, WC1B 5JU UK

**Keywords:** Self-organisation, Emotional problems, Conduct problems, Wellbeing, Life satisfaction, Childhood intelligence

## Abstract

**Background:**

Mental health problems in adolescence are predictive of future mental distress and psychopathology; however, few studies investigated adolescent mental health problems in relation to future mental wellbeing and none with follow-up to older age.

**Aims:**

To test prospective associations between adolescent mental health problems and mental wellbeing and life satisfaction in early old age.

**Methods:**

A total of 1561 men and women were drawn from the Medical Research Council National Survey of Health and Development (the British 1946 birth cohort). Teachers had previously completed rating scales to assess emotional adjustment and behaviours, which allowed us to extract factors of mental health problems measuring self-organisation, behavioural problems, and emotional problems during adolescence. Between the ages of 60–64 years, mental wellbeing was assessed using the Warwick-Edinburgh Mental Well-being Scale (WEMWBS) and life satisfaction was self-reported using the Satisfaction with Life Scale (SWLS).

**Results:**

After controlling for gender, social class of origin, childhood cognitive ability, and educational attainment, adolescent emotional problems were independently inversely associated with mental wellbeing and with life satisfaction. Symptoms of anxiety/depression at 60–64 years explained the association with life satisfaction but not with mental wellbeing. Associations between adolescent self-organisation and conduct problems and mental wellbeing and life satisfaction were of negligible magnitude, but higher childhood cognitive ability significantly predicted poor life satisfaction in early old age.

**Conclusions:**

Adolescent self-organisation and conduct problems may not be predictive of future mental wellbeing and life satisfaction. Adolescent emotional problems may be inversely associated with future wellbeing, and may be associated with lower levels of future life satisfaction through symptoms of anxiety/depression in early old age. Initiatives to prevent and treat emotional problems in adolescence may have long-term benefits which extend into older age.

## Background

Mental disorders are a leading cause of loss of health to disease in middle and high-income countries, and the resulting economic costs can be huge [1]. Mental health is multidimensional, including mental disorders but also positive mental functioning. According to Keyes, there is wide variation in levels of mental health in the general population, with some people ‘flourishing’ (enthusiastic about life and actively engaged with other people), others ‘languishing’ (‘a life of quiet despair’) [2], and the remainder ‘moderately mentally healthy’ [3]. Keyes’ view that mental health should be regarded not just as the absence of mental illness but as a state of complete emotional, psychological, and social wellbeing is part of a growing international interest in what has come to be called positive mental health, often referred to as mental wellbeing [3, 4].

Adolescent mental health problems are risk factors for future mental distress and psychopathology. Adolescent conduct problems are strongly associated with risk of psychiatric disorders, such as depression and substance abuse, in later life [5–8]. Many studies have also reported that adolescent depression is associated with a strong, specific, and direct risk of recurrence in adulthood [9–13]. Recent reports from birth cohort studies have indicated that not only conduct and emotional problems during adolescence, but also low self-organisation (defined in terms of ‘effortful regulation of the self by the self’) [14], may be a significant developmental precursor predicting future mental health problems including substance dependence, depression, and hallucinations [13, 15, 16]. While such empirical evidence leaves no doubt that adolescent mental health problems are associated with poor mental health in later life, to our knowledge no study has investigated adolescent mental health problems in relation to mental wellbeing in later life.

Childhood factors, particularly childhood cognitive ability and early-life socioeconomic position may affect adolescent mental health [17], and educational attainment by early adulthood may be a possible mediator between adolescent mental health problems and future mental wellbeing [6, 9]. Analyses that consider the effects of these variables are needed when examining association between adolescent mental health problems and mental wellbeing in old age. In addition, an analysis that considers mental ill health such as symptoms of anxiety/depression in later life as a confounder is needed to assess whether association between adolescent mental health problems and mental wellbeing in later life are fully explained by mental ill health in later life or whether adolescent mental health problems also have implication for future mental wellbeing.

The current study aims to investigate associations between adolescent mental health problems (lower self-organisation, conduct and emotional problems) and mental wellbeing and life satisfaction in early old age considering the effects of childhood cognitive ability, early-life socioeconomic position, educational attainment and mental ill-health in early old age, using longitudinal data from The Medical Research Council National Survey of Health and Development (NSHD, the 1946 British birth cohort study) which is one of the longest continuously running studies of human development and aging in the world [18].

## Methods

### Participants

The NSHD originally consisted of a socially stratified sample of 5362 singleton children (girls: *n* = 2547, boys: *n* = 2815) born within marriage during 1 week in March 1946 in mainland Britain, and includes regular follow-ups throughout life [18]. Mothers, school teachers and the study members themselves provided information in childhood and adolescence. This study used outcome data from the follow-up at 2006–2010 (when study members were 60–64 years old). In total, 2856 eligible study members (those known to be alive and with a known address in England, Scotland, or Wales) were invited for an assessment at one of six clinical research facilities (CRFs) or, for those unwilling or unable to travel, during a visit from a research nurse at home. Mental wellbeing and life satisfaction were measured using self-report assessments included in this visit. Invitations were not sent to those who were known to have died (*n* = 778), were living abroad (*n* = 570), had previously withdrawn from the study (*n* = 594), or had been lost to follow-up (*n* = 564). A total of 2229 study members out of the 2856 invited (78.0 %, age range 60.3–65.0 years, mean–63.4, SD = 1.1) underwent assessment: 1690 attended the clinic (CRF) and the remaining 539 were seen in their homes [19]; 229 of these were part of a feasibility stage at which mental wellbeing and life satisfaction were not measured. The current study protocol received ethical approval from the Greater Manchester Local Research Ethics Committee for the four English sites; the Scotland A Research Ethics Committee approved the data collection taking place in Edinburgh. Written informed consent was obtained from participants at each stage of the data collection.

### Measures

#### Mental wellbeing and life satisfaction at 60–64 years of age

Mental wellbeing was assessed using the Warwick-Edinburgh mental wellbeing scale (WEMWBS) [20]. This scale was developed to measure a broad conception of mental wellbeing, including positive affect, psychological functioning (autonomy, competence, self-acceptance, personal growth), and interpersonal relationships, and to be suitable for monitoring mental wellbeing at the population level. Confirmatory factor analysis suggests that it measures a single underlying concept [20]. It has been validated on a representative general population sample of adults. The scale consists of 14 positively worded statements. Examples include: ‘I’ve been feeling optimistic about the future’, ‘I’ve been feeling interested in other people’, ‘I’ve been dealing with problems well’, ‘I’ve been feeling good about myself’, ‘I’ve been feeling useful’. For each statement, respondents are asked to indicate which of five options best describes their experience over the last 2 weeks [scores range from none of the time (1) to all of the time (5)]. The overall score is calculated by summing the scores for each item. A higher score indicates a higher level of mental wellbeing. The Cronbach’s alpha for the 14 items from the NSHD used in this study was 0.91, indicating high internal consistency.

The Satisfaction with Life Scale (SWLS) was developed by Diener et al. [21] to capture this cognitive-evaluative component of mental wellbeing and is widely used [22, 23]. There are five items: ‘In most ways my life is close to my ideal’, ‘The conditions of my life are excellent’, ‘I am satisfied with my life’, ‘So far I have got the important things I want in life’, and ‘If I could live my life again, I would change almost nothing’. Respondents are asked to answer each item on a 7-point Likert scale (ranging from strongly disagree to strongly agree). Answers are added together to create a summary score from 5 to 35. A higher score indicates a higher level of satisfaction with life. The Cronbach’s alpha for the five items from the NSHD used in this study was 0.90, indicating high internal consistency.

#### Adolescent mental health problems

Full details of the measure of adolescent mental health problems have been presented elsewhere Xu et al. [24]. School teachers were asked to rate the behaviour of study members on a three-category response scale where they compared each participant’s behaviour to that of ‘a normal child’ at age 13 and again at age 15, using 28 items (see Table 1 in Xu et al. [24] for descriptions of factor loadings and item wordings) that were forerunners of those included in the Rutter A scale [25, 26]. These data were subjected to separate exploratory factor analysis using item response theory methods in the statistical software package Mplus 6.1 [27]. Data were modelled as ordinal variables using weighted least squares means and adjusted variance estimates with a probit link. Examination of scree plots, eigenvalues, and model fit indices suggested a three-factor solution (see Table 1 in Xu et al. [24] for descriptions of factor loadings and item wordings). Specifically, the factor ‘self-organisation’ was identified as a dimension separate from ‘conduct’ (e.g. disobedience, evading truth to keep out of trouble) and ‘emotional’ (e.g. gloomy and sad, extremely fearful) problems. The self-organisation factor, which correlates more with the conduct factor than the emotional factor, was defined by items relating to attitude to work, concentration, neatness in work, and not daydreaming in class [24]. Factor scores were exported for each factor at age 13 and 15 years (scores at these two ages were moderately correlated: self-organisation (r = 0.48), emotional problems (r = 0.41) and conduct problems (r = 0.51), respectively) and summed to create overall scores for these dimensions, with a higher score indicating lower self-organisation and more severe emotional and conduct problems. To facilitate interpretation, the new combined scales were then standardized to form *z*-scores.

**Table 1:** Descriptive statistics for all study variables; the top panel presents means (SD) and the bottom panel presents *n* (%)

	Males (*n* = 735)	Females (*n* = 826)	All (*n* = 1561)
Mental wellbeing (WEMWBS) (range = 21–70)	51.49 (7.57)	51.55 (8.42)	51.52 (8.03)
Life satisfaction (SWLS) (range = 5–35)	26.97 (5.71)	26.42 (6.29)	26.67 (6.03)
Symptoms of anxiety/depression (GHQ-28) (range = 29–87)	43.09 (7.21)	45.78 (8.88)	44.61 (8.34)
Childhood cognitive ability at age 8	90.21 (27.81)	93.01 (26.73)	91.69 (27.27)
Adolescent mental health problems (top quartile)			
Self-organisation	226 (30.7)	164 (19.9)	
Conduct problems	201 (27.3)	189 (22.9)	
Emotional problems	155 (21.1)	234 (28.3)	
Father’s occupational social class			
Manual	395 (53.7)	446 (54.0)	841 (53.9)
Non-manual	340 (46.3)	380 (46.0)	720 (46.1)
Educational attainment by age 26			
No qualifications	235 (32.0)	261 (31.6)	496 (31.8)
Vocational	45 (6.1)	80 (9.7)	125 (8.0)
Ordinary (‘O’ level)	109 (14.8)	216 (26.2)	325 (20.8)
Advanced (‘A’ level)	227 (30.9)	216 (26.2)	443 (28.4)
Higher	119 (16.2)	53 (6.4)	172 (11.0)

#### Covariates

Childhood cognitive ability at age 8 was represented as the sum of four tests of verbal and nonverbal ability devised by the National Foundation for Education Research [28]. These tests include (a) reading comprehension (selecting appropriate words to complete 35 sentences); (b) word reading (ability to read and pronounce 50 words); (c) vocabulary (ability to explain the meaning of 50 words); and (d) picture intelligence, consisting of a 60-item nonverbal reasoning test. We used confirmatory factor analysis to construct a scale summarizing these data. Model fit indices were Chi square = 63.145 with one degree of freedom, RMSEA = 0.121, CFI = 0.994, and TLI = 0.966. Factor scores were computed and then standardized to a mean of zero with a standard deviation of one. Early-life socioeconomic position was assessed using the occupational social class of the father when study members were aged 11 years (or at 4 or 15 years, if this was unknown). Cognitive ability and social class may be selective for adolescent self-organisation, conduct, and emotional problems and were considered as possible confounders in the association between these mental health problems and mental wellbeing at age 60–64. Educational attainment was based on the highest educational qualifications and their training equivalents attained by 26 years of age, and were classified as none, vocational only, ordinary secondary (O levels), advanced secondary (A levels), or degree level or equivalent. Symptoms of anxiety and depression at age 60–64 were assessed by the General Health Questionnaire (GHQ-28) [29]. The GHQ-28 is a self-administered screening questionnaire detecting common mental disorders in the general population. Each item on the questionnaire asks respondents if they have had recent complaints (over the past few weeks) such as losing sleep over worry. Each individual item was scored using a 4-point Likert scale, and a total score was calculated.

### Statistical analysis

Associations between total scores for adolescent self-organisation, conduct, and emotional problems, and total scores for the WEMWBS and SWLS were tested using multivariable regression models for each outcome. The first model also adjusted for each score derived from teacher ratings at ages 13 and 15, and also adjusted for gender. The second model adjusted for father’s social class and childhood cognitive ability. In the third model, we additionally adjusted for educational attainment. In the fourth model, we additionally adjusted for GHQ-28 total score at age 60–64 years. All models were estimated using IBM SPSS version 21.0 for Windows (New York).

## Results

### Descriptive statistics

A total of 1936 study members had complete data on WEMWBS and SWLS and, of these, 1561 had complete data on adolescent mental health and covariates (Table 1). Those with missing outcomes had more adolescent mental health problems (more conduct and emotional problems and lower self-organisation), lower social class assessed by occupational group of father in childhood, lower childhood cognitive ability and lower educational attainment than those who had complete outcome data (All *P* < 0.01). While there were no gender differences in mean WEMWBS or SWLS score (*p* = 0.886 and *p* = 0.072, respectively), female sex was positively associated with higher prevalence within the top quartile of emotional problems (*p* < 0.05) and also negatively associated with lower prevalence within the top quartile of self-organisation and conduct problems (*p* < 0.001 and *p* = 0.001, respectively). WEMWBS and SWLS were moderately positively correlated with each other and moderately negatively correlated with GHQ-28 at age 60–64 (Table 2). Adolescent emotional problems were moderately positively correlated with adolescent self-organisation problems but more weakly correlated with conduct problems.

**Table 2:** Correlation matrix of variables

Variable	1	2	3	4	5	6	7	8	9
1. Mental wellbeing (WEMWBS) at age 60-64	1.00	0.550*	−0.056	−0.018	−0.093*	0.066*	−0.039	0.087*	−0.534*
2. Life-satisfaction (SWLS) at age 60–64	0.550*	1.00	−0.033	−0.020	−0.070*	−0.051	0.026	0.031	−0.482*
3. Adolescent self-organization problems	−0.056	−0.033	1.00	0.584*	0.342*	−0.356*	0.228*	−0.415*	0.047
4. Adolescent emotional problems	−0.018	−0.020	0.584*	1.00	−0.104*	−0.205*	0.135*	−0.280*	0.038
5. Adolescent conduct problems	−0.093*	−0.070*	0.342*	−0.104*	1.00	−0.189*	0.078*	−0.158*	0.068*
6. Childhood cognitive ability at age 8	0.066*	−0.051	−0.356*	−0.205*	−0.189*	1.00	−0.358*	0.580*	−0.040
7. Father’s occupational social class	−0.039	0.026	0.228*	0.135*	0.078*	−0.358*	1.00	−0.443*	0.039
8. Educational attainment by age 26	0.087*	0.031	−0.415*	−0.280*	−0.158*	0.580*	−0.443*	1.00	−0.085*
9. Symptoms of anxiety/depression (GHQ−28) at age 60−64	−0.534*	−0.482*	0.047	0.038	0.068*	−0.040	0.039	−0.085*	1.00

* Correlations are significant at p < 0.01 (two−tailed)

### Adolescent mental health and mental wellbeing in later life

Table 3 shows the multivariable linear regression coefficients for total WEMWBS scores. Model 1 (adolescent mental health variables mutually adjusted, and also adjusted for gender) shows that adolescent emotional problems were independently inversely associated with this outcome, whereas adolescent self-organisation and conduct problems were not significantly associated with mental wellbeing. On the whole these coefficients were not substantially changed by further adjustments for childhood cognitive ability and social class of origin (Model 2), or by the addition of educational attainment (Model 3). Adjustment for GHQ-28 scores at age 60–64 years attenuated but did not fully explain the association between mental wellbeing and emotional problems (Model 4). Higher childhood cognitive ability was associated with higher mental wellbeing in later life (Model 2), although this significant association did not remain after additional adjustment for educational attainment (Model 3). Higher GHQ-28 scores at age 60–64 years was independently inversely associated with mental wellbeing (Model 4).

**Table 3:** Associations between WEMWBS at age 60–64 years and scores for self-organisation, conduct, and emotional problems in adolescence

Independent variables	Model 1		Model 2		Model 3		Model 4	
	Coeff.	p-value	Coeff.	p-value	Coeff.	p-value	Coeff.	p-value
Adolescent mental health								
Self-organisation	−0.017	0.6	0.020	0.6	0.027	0.5	0.033	0.3
Conduct problems	−0.023	0.5	−0.021	0.5	−0.023	0.5	−0.003	0.9
Emotional problems	*−0.093*	*0.001*	*−0.091*	*0.001*	*−0.084*	*0.003*	*−0.052*	*0.034*
Childhood cognitive ability at age 8			*0.058*	*0.035*	0.028	0.4	0.021	0.4
Father’s occupational social class			−0.022	0.4	−0.012	0.7	0.003	0.9
Educational attainment by age 26					0.057	0.079	0.038	0.2
GHQ-28 at age 60–64							*−0.551*	*<0.001*

Variable coefficients (Coeff.) are standardized. * Italics* indicates p < 0.05. Model 1 adjusted for each score derived from teacher ratings at ages 13 and 15 as well as for gender. Model 2 further adjusted for childhood cognitive ability at age 8 and father’s occupational social class. Model 3 further adjusted for educational attainment by age 26. Model 4 further adjusted for GHQ-28 total score at age 60–64

### Adolescent mental health and life satisfaction in later life

Table 4 shows the multivariable linear regression coefficients for total SWLS scores. Model 1 (adolescent mental health variables mutually adjusted, and also adjusted for gender) shows that adolescent emotional problems were independently inversely associated with this outcome, whereas adolescent self-organisation and conduct problems were not significantly associated with life satisfaction. These coefficients were not substantially changed by further adjustments for childhood cognitive ability and social class of origin (Model 2), or by educational attainment (Model 3). However, adjustment for GHQ-28 scores at age 60–64 years fully explained the association between adolescent emotional problems and life satisfaction in later life (Model 4). Higher childhood cognitive ability was itself significantly inversely associated with life satisfaction even after adjustment for all covariates (Model 4). Higher GHQ-28 score at 60–64 years was independently inversely associated with life satisfaction (Model 4).

**Table 4:** Associations between life satisfaction at age 60–64 years and scores for self-organisation, conduct, and emotional problems in adolescence

	Model 1		Model 2		Model 3		Model 4	
Independent variables	Coeff.	p-value	Coeff.	p-value	Coeff.	p-value	Coeff.	p-value
Adolescent mental health								
Self-organisation	0.003	0.9	−0.025	0.5	−0.020	0.6	−0.015	0.6
Conduct problems	−0.037	0.2	−0.026	0.4	−0.025	0.5	−0.002	0.9
Emotional problems	*−0.074*	*0.007*	*−0.068*	*0.016*	*−0.061*	*0.034*	−0.038	0.13
Childhood cognitive ability at age 8			*−0.065*	*0.017*	*−0.088*	*0.003*	*−0.098*	*<0.001*
Father’s occupational social class			0.010	0.7	0.022	0.4	0.033	0.2
Educational attainment by age 26					0.058	0.074	0.044	0.12
GHQ-28 at age 60–64							*−0.493*	*<0.001*

Variable coefficients (Coeff.) are standardized.* Italics* indicates p < 0.05. Model 1 adjusted for each score derived from teacher ratings at ages 13 and 15 as well as for gender. Model 2 further adjusted for childhood cognitive ability at age 8 and father’s occupational social class. Model 3 further adjusted for educational attainment by age 26. Model 4 further adjusted for GHQ-28 total score at age 60–64

## Discussion

Using longitudinal data from a national birth cohort, we found that emotional problems in adolescence predicted mental wellbeing and life satisfaction in early old age, after controlling for gender, social class of origin, childhood intelligence, and educational attainment. The association with life satisfaction may operate through symptoms of anxiety/depression in early old age. We did not find any significant associations between adolescent self-organisation or conduct problems and future mental wellbeing or life satisfaction.

Empirical evidence leaves little doubt that adolescent emotional problems are associated with risk of future mental ill-health such as depression [9, 30]. Our current study revealed that adolescent emotional problems may also affect mental wellbeing (positive mental health based on an instrument that captures positive mental functioning and satisfying personal relationships as well as positive affect and the cognitive-evaluative dimension of wellbeing) in early old age even after controlling the effect of concurrent symptoms of anxiety/depression, suggesting that a pathway which was not fully explained by symptoms of anxiety/depression may be operating. On the other hand, in our study, a significant association between adolescent emotional problems and life satisfaction measured by SWLS in early old age was fully attenuated after adding concurrent symptoms of anxiety/depression. Life satisfaction has been defined as an overall assessment of individual’s quality of life according to his/her chosen criteria [21]. It may be reasoned that mental health problems such as depression may play important role in the cognitive-evaluative process of life satisfaction. In contrast, data from the 1970 British Cohort Study show that greater conduct problems in childhood and adolescence were associated with lower life satisfaction at age 34 [31]. Explanation for this discrepancy may possibly be due to the ages at which outcomes were assessed, the different life satisfaction or conduct instruments employed, or different long-term implications of conduct problems during the adult lives of those born more recently.

Some birth cohort studies have reported that poor self-organisation in adolescence might be a significant developmental precursor predicting a broad range of adverse outcomes in later life including educational and occupational underachievement, poor physical health, and trouble with the criminal justice system [13, 15]. However, these studies have not considered the effects of both conduct and emotional problems in adolescence as confounders. Our previous study revealed that poor self-organisation in adolescence was a risk factor for future psychopathology, such as hallucination, after controlling for the effects of adolescent conduct and emotional problems [16]. On the other hand, our current results suggest that self-organisation in adolescence was not associated with mental wellbeing and life satisfaction after controlling for the effects of adolescent conduct and emotional problems. Together, these pieces of evidence suggest that lack of self-organisation in adolescence might be a driver for later mental illness, but that self-organisation is not related to later positive mental wellbeing once we consider the effects of past conduct and emotional problems.

Our current results indicate that conduct problems in adolescence were not associated with mental wellbeing or life satisfaction in later life (after controlling for the effects of emotional problems and self-organisation in adolescence). Among teenagers, rates of comorbidity between depression and conduct problems are high [32]. Conduct problems are strongly associated with early-onset depression, the most common pattern being that conduct problems precede the expression of affective symptomatology [9]. Therefore, it is necessary to consider the mutual effects of these two factors when investigating the associations between conduct and emotional problems in adolescence and future outcomes.

It is also worth noting that childhood cognitive ability was associated with poorer life satisfaction in early old age after adjustment for social class in origin, educational attainment, and symptoms of anxiety/depression in later life. Analysis of a younger birth cohort to age 34 showed a positive correlation between life satisfaction and childhood and adolescent cognitive ability, though this was smaller in magnitude than the correlations between life satisfaction and either emotional or conduct problems in childhood and adolescence [31]. In contrast, the Lothian birth cohort 1921 study, which consisted of 550 older adults, showed that correlations between IQ at age 11 and the life satisfaction measured by SWLS at age 79 were null (*r* = 0.00) [33], although the study did not control for adolescent mental health, educational attainment, or depression in later life. In order to explain the absence of this relation, the authors refer to the fact that higher ability is equally likely to lead to positive and negative outcomes (e.g. increasing one’s resources through entry to better employment, and an awareness of alternative lifestyles or striving for greater achievement, respectively), which may be involved in measuring subjective wellbeing [34]. Our results suggest that higher childhood cognitive ability is more likely to lead to negative outcomes when considering the additional effects of adolescent mental health, educational attainment, and symptoms of anxiety/depression in later life.

Strengths of this study include the use of a national population-based sample, the availability of independent teacher ratings for adolescent mental health, and a comprehensive range of potential explanatory variables, including cognitive ability measured during childhood.

Against these strengths we acknowledge some limitations. A limitation of this study is attrition of survey members over the 60 year follow-up period. Typically for longitudinal health-related studies, there was disproportional loss to follow-up in the NSHD for those who were relatively less socially advantaged, had poorer adolescent mental health, and who had lower childhood cognitive ability. However, while this might have led to some degree of underestimation of association strengths, we have no reason to believe that this would have influenced the *pattern* of these associations. As there were no data on mental wellbeing and life-satisfaction in adolescence in NSHD, the autoregressive effect of them could not be controlled in our analysis. It therefore remains unclear whether adolescent emotional problems predict mental wellbeing once pre-existing level of mental wellbeing is controlled. The NSHD data contain only teachers’ assessments of the children’s emotional adjustment and behaviours, with no information from the parents or the children themselves. However, teachers can make an important contribution to the identification of adolescent behavioural problems, which is often missed when children or parents report on behaviour [35]. We used data of teachers’ assessments of the children’s emotional adjustment and behaviours collected almost 50 years ago to make a recently-identified construct, self-organisation. In our study, adolescent self-organisation was defined through secondary analysis by a relatively small number of items, rather than by an instrument specifically designed to capture this construct; however, the psychometric techniques used to distinguish it from adolescent emotional and conduct problems were rigorous, and the resulting measure was found to have discriminant properties [24].

## Conclusions

In a prospective UK birth cohort study we found that adolescent emotional problems may be negatively associated with future mental wellbeing and life satisfaction, partly through symptoms of anxiety/depression. Adolescent self-organisation and conduct problems did not show this pattern. Our results suggest that prevention of and interventions for emotional problems during development might have important, long-reaching consequences for individuals’ future mental wellbeing.
